# Brain fMRI study in elderly BPPV patients with comorbid anxiety and depression

**DOI:** 10.1186/s12883-026-04886-6

**Published:** 2026-04-22

**Authors:** Fangying Tao, Yuqing Liu, Kejie Yang, Hui Yang, Xue Yang, Ye Liu, Zhipeng Wang, Zuwei Cao

**Affiliations:** 1 Department of Hearing Rehabilitation Research Center, Guizhou Provincial People’s Hospital, Guiyang, 550002 China; 2 Department of Otolaryngology, Guizhou Branch of Shanghai Children’s Medical Center, Shanghai Jiao Tong University School of Medicine, Guiyang, 550081 China; 3 Department of Psychological, Guizhou Provincial People’s Hospital, Guiyang, 550002 China; 4 Department of Otolaryngology, Guizhou Provincial People’s Hospital, Guiyang, 550002 China

**Keywords:** Benign paroxysmal positional vertigo (BPPV), Functional magnetic resonance imaging(fMRI), Amplitude of low-frequency fluctuations (ALFF), Functional connectivity (FC), Hippocampus, Anxiety/Depression

## Abstract

**Background:**

A significant proportion of benign paroxysmal positional vertigo (BPPV) patients suffer from anxiety and depression, severely affecting their quality of life. Functional magnetic resonance imaging (fMRI), as a non-invasive technique, has been widely applied in exploring the pathophysiology of neuropsychiatric disorders. This study used fMRI-based Amplitude of low-frequency fluctuations (ALFF) analysis and seed-based functional connectivity (FC) mapping to investigate brain activity and connectivity changes in elderly BPPV patients, aiming to clarify functional brain alterations associated with anxiety and depression.

**Methods:**

Elderly BPPV patients with anxiety/depression (BPPV, *n* = 25) and age-matched healthy control subjects (HCs, *n* = 20) underwent resting-state fMRI scanning. The ALFF was calculated to observe the intrinsic brain activity across all brain voxels. Brain regions with altered ALFF were further selected as seeds for FC analysis. Correlation analysis was performed between ALFF/FC and clinical parameters in BPPV patients.

**Results:**

Compared with HCs, BPPV patients exhibited significantly increased ALFF in the bilateral hippocampal regions and the left parahippocampal area. When the bilateral hippocampal regions and the left parahippocampal area were selected as seed regions, significantly decreased in FC were observed in the left cerebellar anterior lobe. In addition, the ALFF values in the bilateral hippocampal regions were positively correlated with the severity of anxiety, depression, and vertigo in BPPV patients. The ROC curves of the altered brain regions indicated great accuracy in distinguishing between BPPV patients and HCs.

**Conclusions:**

The hippocampal region, a critical area in regulating emotional and cognitive functions, exhibited increased neural activity and disrupted cerebellar connectivity, which may exacerbate anxiety and depression in elderly BPPV patients. This study provides preliminary insights into the potential neural mechanisms related to mood and cognitive dysfunctions in BPPV patients.

## Background

Benign paroxysmal positional vertigo (BPPV) is the most prevalent vestibular disorder, characterized by transient yet severe episodes of vertigo triggered by changes in head position. Epidemiological studies worldwide indicate that BPPV affects approximately 10% of the general population [1]. The incidence of BPPV increases significantly with age, becoming the leading cause of dizziness or vertigo in older adults, accounting for about one-third of all diagnosed cases [2]. Additionally, BPPV has a high recurrence rate, with approximately 50% of patients experiencing a recurrence within 10 years of follow-up [3]. Elderly patients with BPPV are at an increased risk of falls and are particularly vulnerable to developing fear, anxiety, and depression. These emotional disturbances can lead to both physical and mental functional impairments, further exacerbating the overall impact on their quality of life and contributing significantly to the economic and social burden [4, 5].

Critically, BPPV extends its effects beyond the peripheral vestibular system. Substantial evidence clearly indicates that anxiety and depression are significant adverse prognostic factors, influencing the clinical progression and recovery of vertigo disorders [4, 6]. The relationship between these conditions is often bidirectional: recurrent vertigo episodes can trigger or exacerbate anxiety and depression, which, in turn, may amplify dizziness perception and hinder recovery. This creates a detrimental cycle that complicates treatment [7, 8]. Notably, research has shown that anxiety and depression can contribute to persistent residual dizziness in elderly BPPV patients, even after successful resolution of canalithiasis through repositioning maneuvers [8]. In some cases, this may progress to chronic conditions such as Persistent Postural-Perceptual Dizziness (PPPD), characterized by ongoing non-vertiginous dizziness and unsteadiness, which are exacerbated by upright posture and complex visual stimuli [7, 8]. These comorbidities severely impact patients’ quality of life, reduce work efficiency, and elevate healthcare costs [9].

The precise neural pathways through which a peripheral vestibular disturbance, such as BPPV, contributes to affective disorders remain an area of ongoing investigation. However, neuroimaging studies have provided key insights, revealing alterations in brain networks that are crucial for balance, emotion, and anxiety regulation. Previous research has identified both functional and structural changes within the prefrontal-limbic network, a critical circuit for emotional regulation [9]. Moreover, studies on middle-aged and elderly individuals with vestibular dysfunction have reported white matter abnormalities in the brain and limbic regions, which are also linked to depression and anxiety. These findings suggest a shared neural substrate that underlies both vestibular and emotional processing [8, 10].

Advanced neuroimaging techniques have played a crucial role in elucidating the neural correlates of vestibular dysfunction and affective disorders. Diffusion Tensor Imaging (DTI) has identified microstructural white matter deficits within limbic pathways in patients with comorbid depression and vestibular dysfunction. For example, Chen et al. demonstrated reduced fractional anisotropy in a circuit involving the medial prefrontal cortex, hippocampus, amygdala, and thalamus, indicating compromised white matter integrity and disrupted neural communication [10]. In addition, fMRI has revealed altered functional connectivity within the default mode network and limbic system in these patients, which may underlie the pathophysiology of comorbid anxiety and depression [11]. Notably, these neuroimaging biomarkers appear to exhibit neuroplastic potential, with previous studies confirming that interventions such as cognitive behavioral therapy can induce structural and functional brain adaptations [12]. The interaction between neuroinflammation and these imaging abnormalities is also garnering increasing attention. It has been suggested that peripheral inflammatory mediators might compromise blood-brain barrier integrity, exacerbating emotional comorbidities by inhibiting hippocampal neurogenesis, impairing synaptic plasticity, and promoting white matter degeneration [13].

Resting-state fMRI (rs-fMRI) has emerged as a leading non-invasive technique for investigating the pathogenesis of neurological and neuropsychiatric disorders. It identifies variations in spontaneous neural activity by measuring the blood oxygen level-dependent (BOLD) signal. Two key analytical methods in rs-fMRI are ALFF and FC. ALFF quantifies the intensity of spontaneous neural activity at the voxel level, providing a direct measure of local neuronal baseline activity [14, 15]. In contrast, seed-based FC assesses the temporal correlation of BOLD signals between distinct brain regions, allowing for the mapping of functional networks [15]. While FC requires a priori selection of regions of interest, ALFF offers the unique advantage of detecting localized neural activity abnormalities without such assumptions, making it particularly valuable for exploratory studies in disorders with complex and poorly defined neural substrates [14]. ALFF has proven sensitive in detecting subtle neural dysfunction in various sensory-affective and inflammatory conditions, even during clinical remission, positioning it as a reliable standalone biomarker [16, 17].

Despite these advances, the specific pathophysiology of anxiety and depression in the context of peripheral vertigo, particularly BPPV, has rarely been explored using these tools. A limited number of studies have initiated this research; for example, one rs-fMRI study on BPPV patients with residual dizziness found that altered functional connectivity in the parietal operculum (OP2) might serve as a diagnostic biomarker [15]. However, a comprehensive characterization of localized neural activity and its connection to broader network dysfunction in elderly BPPV patients with affective comorbidities remains lacking.

Therefore, based on current research trends, this study begins with the clinical observation of BPPV comorbid with anxiety and depression. The study employs ALFF and seed-based FC methods to comprehensively explore both local brain activity and inter-regional functional connectivity, aiming to provide potential insights into brain function changes in BPPV. We hypothesize that brain regions involved in emotional and cognitive functions may exhibit abnormal brain activity and functional connectivity in BPPV patients, and that these altered brain regions may be associated with anxiety and depression.

## Materials and methods

### Participants

**Fig. 1:**
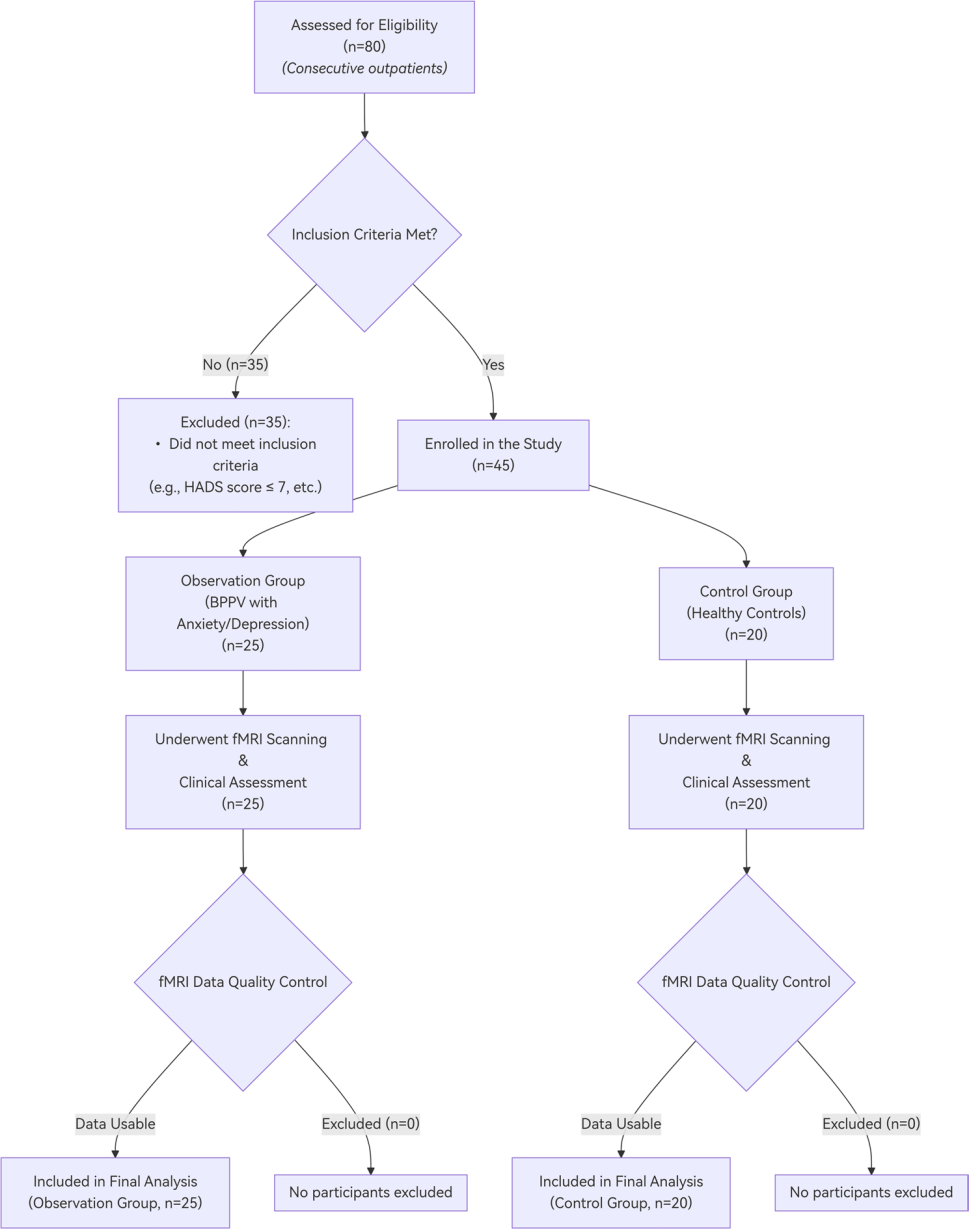
The STROBE flowchart of this study

This experiment included 25 elderly patients (aged 60–85 years) diagnosed with BPPV who were treated at the Hearing Rehabilitation Research Center of Guizhou Provincial People’s Hospital, China, between Jan and Dec 2023. The patients are all from Guizhou Province, China. All patients participated voluntarily and met the following inclusion criteria. The top 25 patients were selected (Detailed information is provided in Fig. 1). The patients’ disease duration varied widely, spanning from 1 day to 2 years, and exhibited a large standard deviation (details are presented in Table 1). This study collected data on the age, gender, educational level, Hospital Anxiety and Depression Scale (HADS) scores, Dizziness Handicap Inventory (DHI) scores, and Vestibular Symptom Index (VSI) scores of the participants. The diagnostic criteria for BPPV were based on previous clinical guidelines [18, 19]. The diagnosis was primarily determined using the patient’s history and positional test results. Patients potentially suffering from anxiety or depression underwent screening with the HADS. Individuals exhibiting abnormal HADS scores were assigned to the observation group. The criteria for inclusion in this group were as follows: (1) A DHI score indicating mild or moderate abnormalities or worse [20]; (2)A VSI score, where 0 indicates complete normality and 10 indicates intolerable symptoms, with higher scores correlating with more severe symptoms [21]; (3) A HADS score greater than 7, which was defined as abnormal [22].

**Table 1:** Demographic and clinical parameters

	Eldly BPPV(patients)	HCs	Statistics	*P*-value
	*n* = 25	*n* = 20		
Demographic characteristics				
Gender: Men (%)	12 (48%)	9 (45%)	χ = 0.0	1^b^
Age (year)	73.88 ± 5.93	74.57 ± 5.69	*t* = − 0.398	0.693^a^
Education (year)	14.00 ± 3.15	14.16 ± 2.20	*t* = − 0.200	0.843^a^
Clinical characteristics				
dizziness	20(80%)	N/A	N/A	N/A
vertigo	25(100%)	N/A	N/A	N/A
Nausea or (and) vomiting	18(72%)	N/A	N/A	N/A
Fall down	12 (48%)		N/A	N/A
Diarrhea	5(25%)	N/A	N/A	N/A
Nystagmus	25(100%)	N/A	N/A	N/A
Questionnaires				
HADS total scores	12.96 ± 3.80	9.90 ± 2.86	*t* = 3.08	0.002^a^
HADS-A scores	6.80 ± 1.69	5.455 ± 1.90	*t* = 2.48	0.014^a^
HADS-D scores	6.19 ± 2.15	4.70 ± 2.34	*t* = 2.20	0.030 ^a^
DHI	26.76 ± 6.26	N/A	N/A	N/A
VSI	19.88 ± 6.13	N/A	N/A	N/A

Data are expressed as mean ± standard deviation. For (a), *P* values are derived using a two-sample t-test, and for (b), a chi-square test is applied

*N/A* not applicable,  *HADS* Hospital Anxiety and Depression Scale, *HADS-A* Anxiety subscale of HADS, *HADS-D* Depression subscale of HADS, *DHI* Dizziness Handicap Inventory, *VSI* Vestibular Symptom Index

We selected 20 healthy elderly volunteers who underwent a hearing health checkup in our department concurrently, had no BPPV, were not anxious or depressed (HADS-A or HADS-D score < 14 points), and were gender and age-matched with the study group. The exclusion criteria were as follows: (1) Severe anxiety or depression (score > 14 points); (2) Previous history of brain surgery, presence of a tumor, or a neuropsychiatric condition; (3) Background of drug or alcohol misuse; (4) Left-handedness: Left-handed participants were excluded to minimize the potential confounding effects of inter-individual variability in cerebral lateralization on emotion-related brain networks, in line with standard practices in neuroimaging research; (5) Inadvisable for MRI. This study was authorized by the Ethical Review Board of the Guizhou Provincial People’s Hospital (No: (2023) 267). All participants signed the informed consent form. We collected Population Characteristics, including gender, age, disease duration, clinical symptoms, medical history, surgical history, and complications. We utilized the DHI score in combination with symptom assessment to evaluate the severity of BPPV dizziness/vertigo. Additionally, we collected the HADS and VSI scores for each research subject.

The MRI screening was performed utilizing a 3.0 T GE scanner (Model: Discovery MR750) at Guizhou Provincial People’s Hospital. Participants were directed to keep their eyes shut and remain conscious throughout the scanning process, while also being advised to refrain from deep thinking or forceful breathing. To reduce head motion and ambient noise, earplugs and foam padding were utilized during the procedure. A gradient echo plane imaging (EPI) sequence was utilized to obtain 250 cubic centimeters of rs-fMRI in the axial plane. The scan parameters for the 3D-T1WI sequence were as follows: TR = 8.5 ms, TE = 3.2 ms, TI = 450 ms, flip angle = 15°, field of view = 256 × 256 mm, matrix = 256 × 256, and slice thickness = 1 mm. Resting-state fMRI used a gradient echo planar imaging sequence with the following parameters: TR = 2000 ms, TE = 30 ms, slice thickness = 3 mm (no gap), number of slices = 50, field of view = 256 × 256 mm, matrix = 64 × 64, voxel size = 3 × 3 × 3 mm, flip angle = 90°, and 150 time points acquired.

### Preprocessing of data

Resting-state BOLD data were preprocessed using the Data Processing and Analysis for Brain Imaging (DPABI) toolbox, which is based on Statistical Parametric Mapping 12 (SPM12). The pre-processing steps encompassed: discarding the initial 10 volumes per participant to mitigate signal instability; slice timing correction and realignment to address head motion, participants exhibiting circular or translational motion greater than 2 degrees or 2 millimeters were excluded; spatial normalization to the Montreal Neurological Institute brain template via the DARTEL method, followed by resampling to 3-millimeter voxels; smoothing with a 6-millimeter full-width-at-half-maximum Gaussian kernel; correction for nuisance variables, such as linear trend, fluid signal in the cerebrospinal space, signal from the white matter, and Friston’s 24-head movement parameter; and band-pass filtering was applied within the 0.01 to 0.08 Hz range to remove physiological noise.

### ALFF and FC assessments

The ALFF and FC analyses were performed using REST software. For the ALFF analysis, after preprocessing, a Fast Fourier Transform (FFT) was applied to convert the time series into the frequency domain. The square root of the power spectrum was then calculated, and the mean square root across the frequency range of 0.01 to 0.08 Hz was computed to derive the ALFF. To reduce global effects, the ALFF values were normalized by dividing them by the global mean ALFF. The analysis was performed on a voxel-wise basis across the entire brain to examine regional variations. Based on the ALFF results, pathological brain regions were identified and selected as seed regions. A 5 mm radius around the peak MNI coordinates was used as the region of interest (ROI) for seed-based FC analysis. Pearson’s correlation coefficients were calculated between the time courses of seed regions and the time series of all brain voxels. Fisher’s z-transformation was applied to the Pearson correlation coefficients to generate a distribution approximating normality, which was used for subsequent statistical analysis.

### Statistical analysis

SPSS 26.0 was employed to analyze the demographic and clinical data. Two-sample t-tests were used for continuous variables, and chi-square tests for proportions. Amplitude differences in ALFF and FC across voxels were assessed using SPM12, adjusting for gender and age as covariates to control for confounding factors. Multiple comparisons were corrected with a cluster-based FDR method (*P* = 0.001, corrected *P* < 0.05). Spearman correlation was applied to examine the correlation between non-normally distributed HADS and DHI scores and ALFF, FC, and VSI. Pearson correlation assessed the association of normally distributed HADS-D and HADS-A subscales with ALFF and FC. ROC curves evaluated the diagnostic utility of ALFF and FC in differentiating elderly BPPV patients with abnormal brain regions from HCs.

## Results

### Demographics and clinical characteristics

There was no significant difference in gender and age between the elderly BPPV patients and the healthy control group(χ2 = 0.000, *p* = 1.000)(t=-0.398, *p* > 0.050); nevertheless, significant differences were observed in the total HADS score (t = 7.050, *p* = 0.002), HADS-A (t = 9.320, *P* = 0.014), and HADS-D (t = 8.740, *p* = 0.050) scores. There was no statistically significant difference in the education level between the two groups (t=-0.200, *p* > 0.05). Detailed information is provided in Table 1.

### ALFF and seed-based FC analyses

**Fig. 2:**
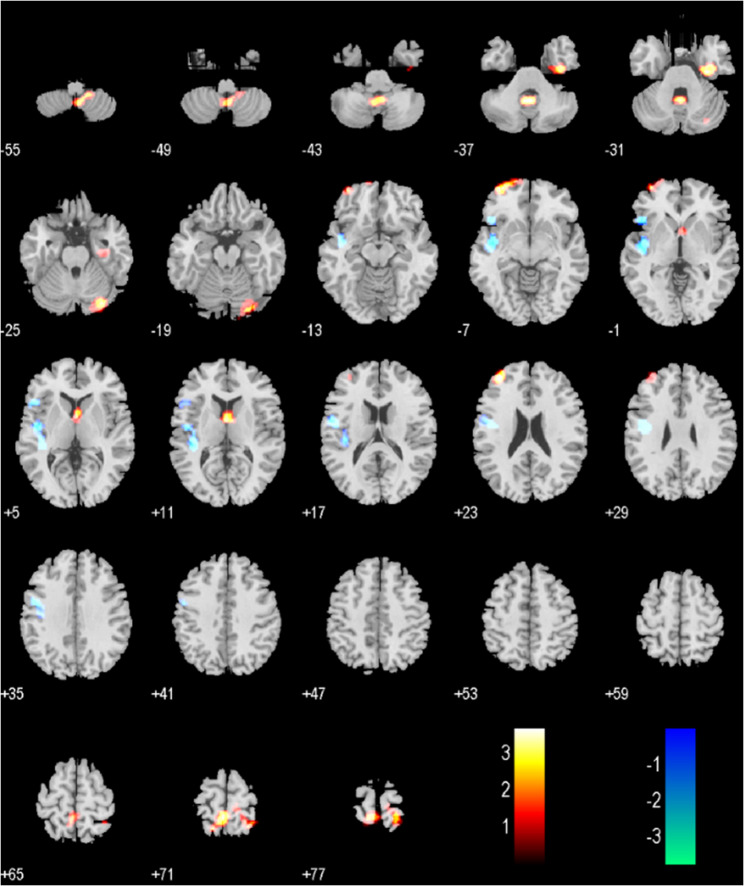
Differences in ALFF between elderly BPPV patients and HCs. Notes: Elderly BPPV patients exhibited significantly higher ALFF in both hippocampi, extending into the left parahippocampal gyrus compared to HCs(*P* < 0.05, FDR corrected)

**Fig. 3:**
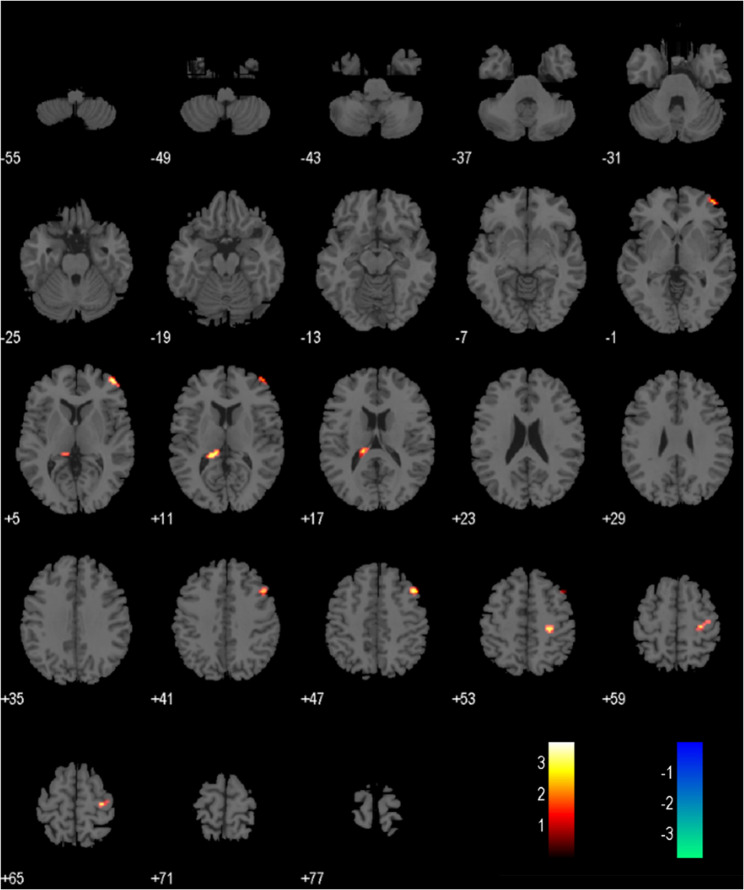
Differences in FC between elderly BPPV patients and HCs. Notes: Compared to HCs, elderly BPPV patients showed significantly enhanced FC between the bilateral hippocampal seed regions and the left anterior cerebellum(*P* < 0.05, FDR corrected)

Compared to the healthy control group, the brain regions showed significantly increased ALFF in elderly BPPV patients were the left hippocampus (cluster size: 102 voxels, t = 4.2312, *p* < 0.050, FDR corrected), the right hippocampus (cluster size: 132 voxels, t = 3.2841, *p* < 0.050, FDR corrected), and the left parahippocampal region (cluster size: 78 voxels, t = 5.4328, *p* < 0.050, FDR corrected). No brain regions showed decreased ALFF (Fig. 2; Table 2). Based on the ALFF analysis results, the left hippocampus, right hippocampus, and left parahippocampal region were selected as seed regions. The functional connectivity between these seed regions and all other voxels in the brain was then calculated. Compared to the healthy control group, the elderly BPPV group showed significantly decreased functional connectivity in the left anterior cerebellum (cluster size: 30 voxels, t= -4.5054, *p* < 0.050, FDR corrected) (Fig. 3; Table 2).

**Table 2:** Differences in ALFF and FC values between Eldly BPPV patients and HCs

Brain regions	Peak MNI coordinates			cluster size	t-Value
	X	Y	Z		
ALFF differences					
Bilateral hippocampals extending to left parahippocampal	-33	60	-6	86	3.414
FC differences					
right cerebellar anterior lobe	27	-27	51	30	-323.505

The x, y, and z coordinates represent the peak voxel locations in standard MNI space. FDR correction was applied at the cluster level with *P* < 0.05. MNI: Montreal Neurological Institute

### Correlation analysis

**Fig. 4:**
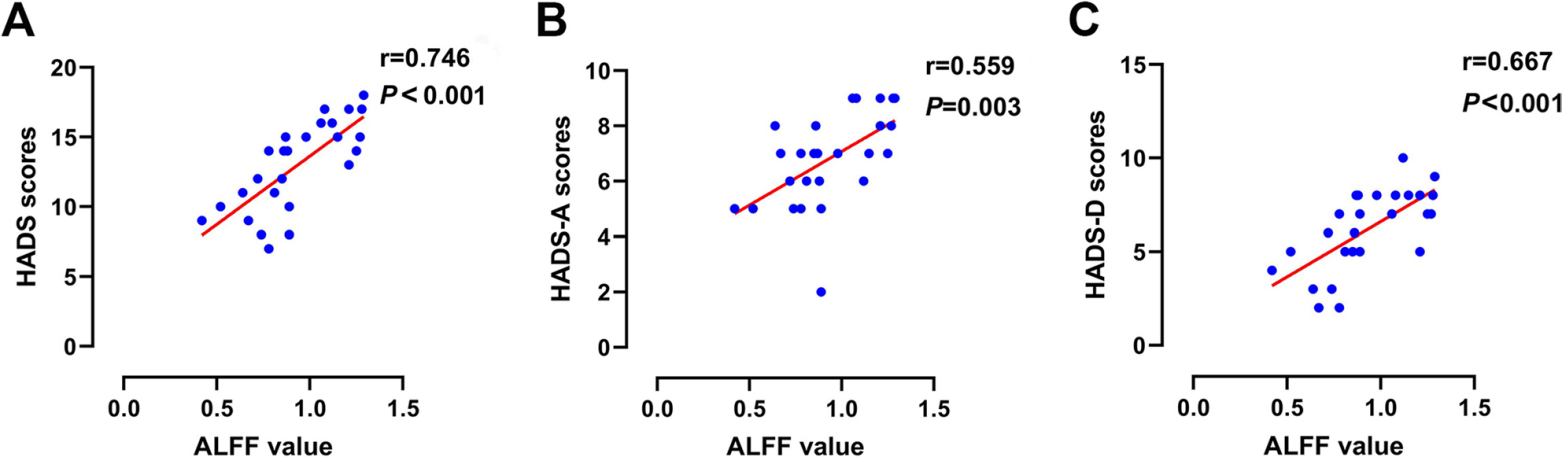
Correlations between the ALFF value in the bilateral hippocampal regions and clinical assessments. Notes: **A**: The ALFF values in the hippocampal region are positively correlated with the HADS score; **B**: The ALFF values in the hippocampal region are positively correlated with the HADS-A score; **C**: The ALFF values in the hippocampal region are positively correlated with the HADS-D score

**Fig. 5:**
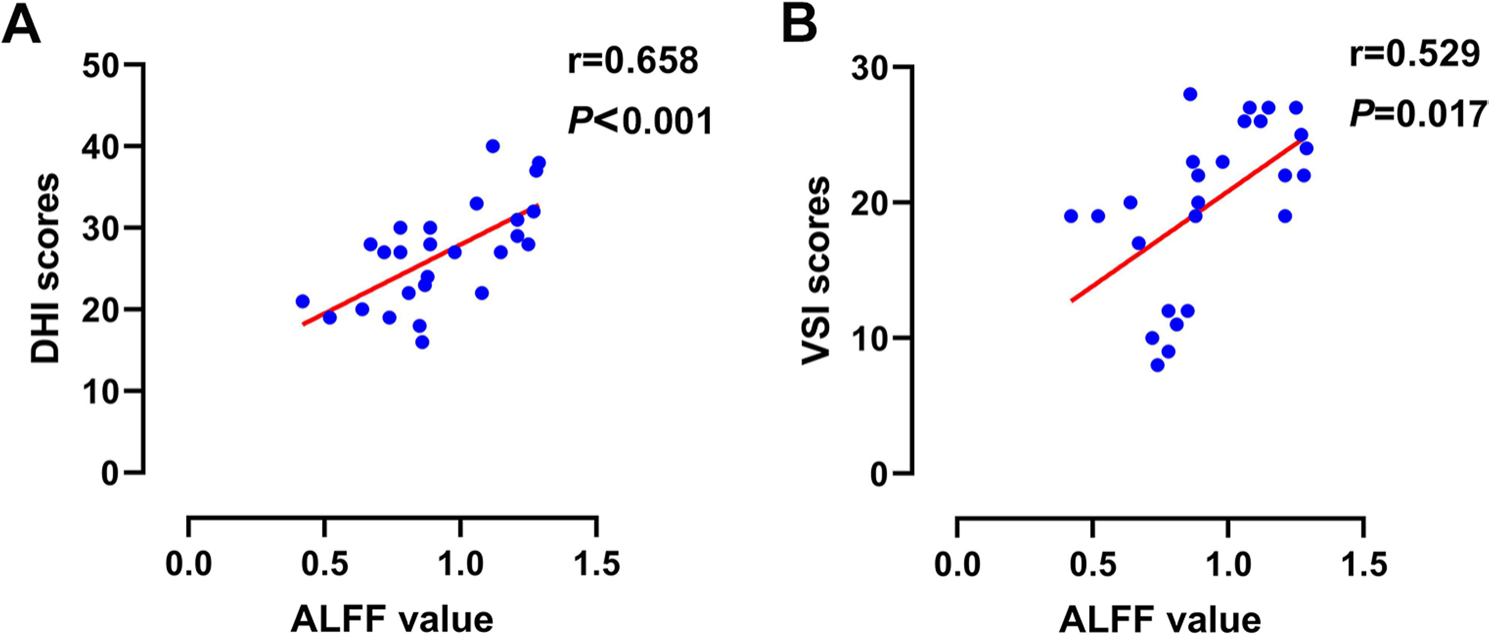
Correlations between the ALFF value in the bilateral hippocampal regions and DHI/VSI. Notes: **A**: The ALFF values in the hippocampal region are positively correlated with the DHI score; **B**: The ALFF values in the hippocampal region are positively correlated with the VSI score

In elderly BPPV patients, A positive correlation was identified between hippocampal region ALFF and HADS scores (*R* = 0.746, *p* < 0.001, Fig. 4a); we found that the ALFF in the hippocampal region was positively associated with the HADS-A score (*R* = 0.559, *p* = 0.003, Fig. 4b), the HADS-D score (*R* = 0.667, *p* < 0.001, Fig. 4c), the DHI (*R* = 0.658, *p* < 0.001, Fig. 5a). Additionally, a significant relationship was observed between the ALFF and the VSI score (*R* = 0.529, *p* = 0.017, Fig. 5b), while no significant correlation was found between FC changes in the left cerebellar anterior lobe and clinical indicators, including the HADS scores, DHI, and VSI.

### ROC analysis

**Fig. 6:**
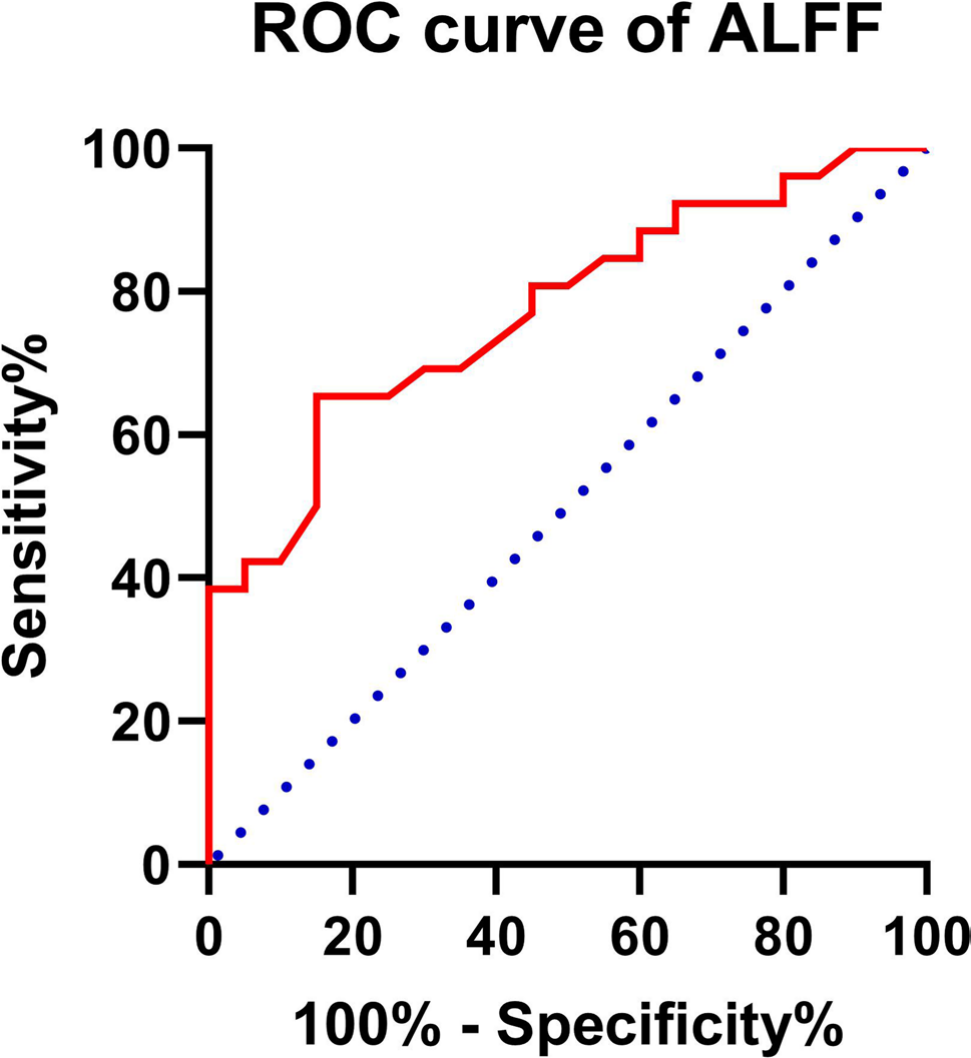
The ROC curve analysis for abnormal brain regions. Notes: The AUC for ALFF in the bilateral hippocampus was 0.774 (*P* = 0.00032; 95% CI: 0.8328–1.0303). ROC refers to receiver operating characteristic, AUC to the area under the curve, and CI to the confidence interval

This study performed ROC curve analyses on the average ALFF and FC values of specific brain regions to identify possible neuroimaging markers that could distinguish elderly BPPV patients with anxiety and depression from HCs. Diagnostic accuracy was determined by the area under the curve (AUC). The AUC for the ALFF value in the bilateral hippocampal regions were 0.774 (Fig. 6), correspondingly, demonstrating the effective distinction of elderly BPPV patients from healthy controls.

## Discussion

This rs-fMRI study investigated the neural correlates of comorbid anxiety and depression in elderly patients with BPPV. Our main findings revealed significantly increased ALFF in the hippocampus and parahippocampal gyrus, along with disrupted FC between these regions and the left cerebellar anterior lobe in patients compared to matched healthy controls. These neuroimaging metrics not only exhibited strong discriminatory power in distinguishing the groups but also correlated positively with the severity of anxiety, depression, and dizziness symptoms. These results suggest that the affective comorbidity frequently observed in BPPV is associated with distinct patterns of localized brain hyperactivity and widespread network dysfunction, specifically within a critical vestibulo-limbic-cerebellar circuitry.

Our findings must be interpreted within the methodological context of a cross-sectional observational study. We explicitly refrain from making causal claims and instead propose that hippocampal hyperactivity represents a significant neural association and a plausible correlate of the comorbid condition. This interpretation is strongly supported by transdiagnostic research which consistently identifies hippocampal alterations as a common feature across mood and anxiety disorders, irrespective of their primary etiology [23]. The chronic, unpredictable, and disabling nature of vertigo attacks in BPPV can be conceptualized as a persistent stressor. As previous studies have confirmed that stress and illness progression are linked to hippocampal changes in depression [24], we speculate that the vestibular distress in BPPV may contribute to a similar maladaptive neuroplasticity, leading to the observed local hyperactivity. This activity may reflect a failure of inhibitory control or a compensatory mechanism, ultimately manifesting clinically as anxiety and depression. Moreover, the correlation between the increased ALFF values in the bilateral hippocampus and the left parahippocampal region with dizziness symptoms, as assessed by the DHI, supports the hypothesis that these brain areas may be implicated in the sensory and emotional processing of vertigo. Similarly, the discovered FC disruption between the hippocampus and the cerebellar anterior lobe points to a broader network dysfunction. The cerebellum, through its extensive connections to the limbic system and association cortices, is now unequivocally recognized as a key node in the distributed neural circuits governing cognition and affect [25–27]. Therefore, the altered FC observed herein likely reflects a disorder or impediment in the coordinated functioning of a network that processes both the sensory-vestibular and the emotional-cognitive dimensions of the BPPV experience.

Our findings have considerable clinical implications for the management of elderly BPPV patients. The significant correlation between elevated hippocampal ALFF and the severity of both vertigo and mood symptoms suggests that this neurobiological marker could eventually serve as an objective tool for identifying a neuropsychiatric subtype of BPPV patients who are at a higher risk for significant psychological morbidity. This reinforces the conclusion of epidemiological and meta-analytic work that routine screening for anxiety and depression in BPPV patients is imperative [28, 29]. Our study provides a potential biological basis for this comorbidity, which could help in destigmatizing these symptoms for patients and encouraging clinicians to adopt a more holistic, biopsychosocial approach. Furthermore, the involvement of the cerebellum, a region intimately linked to vestibular function, offers a novel perspective. Beyond successful canalith repositioning maneuvers, the management plan for patients with this neurobiological profile could be augmented with interventions specifically targeting mood and anxiety, such as cognitive-behavioral therapy or pharmacotherapy. Effective management of the affective symptoms could, by reducing central stress and maladaptive plasticity, positively influence the functional connectivity within this network and potentially improve vestibular outcomes and reduce recurrence rates. Future research focused on whether these ALFF and FC values normalize following successful treatment will be crucial in determining their utility as prognostic biomarkers.

While our study delineates associations between brain function and clinical symptoms, the fundamental molecular mechanisms driving these functional alterations remain unexplored. Considering extant literature, we hypothesize that neuroinflammatory pathways may constitute one plausible, though highly speculative, mechanism bridging chronic vestibular distress and the observed neural dysfunction. It is imperative to state that this study did not measure any inflammatory biomarkers; therefore, this discussion is intentionally conjectural and presented solely to generate testable hypotheses. This speculation is grounded on several pillars. First, the hippocampus is exquisitely vulnerable to the effects of inflammation and stress [24]. Second, transdiagnostic reviews suggest that pro-inflammatory processes may mediate the neurobiological alterations common across mood and anxiety disorders [23]. We propose that the persistent vestibular dysfunction in BPPV acts as a chronic stressor, potentially activating a stress response and triggering the release of pro-inflammatory cytokines [10]. These signaling molecules could modulate synaptic plasticity and disrupt functional connectivity in vulnerable brain regions such as the hippocampus and cerebellum [25, 30], which are known to be involved in both vestibular processing and affective regulation. Thus, while inflammatory mechanisms are not a finding of our study, they present a framework for future research.

### Limitation

This study has limitations. First, the relatively small sample size and the exclusive inclusion of elderly BPPV patients may limit the applicability of the findings to the broader BPPV population. Previous research has shown that the prevalence of psychiatric disorders, such as anxiety and depression, is higher among elderly BPPV patients compared to healthy controls [1–3], which is why this study focused solely on elderly patients. However, this could introduce bias, as the findings may not be representative of BPPV patients from other age groups. Future research should include larger and more diverse cohorts, encompassing BPPV patients from different age groups, to improve the accuracy of the results. Lastly, to gain a more comprehensive understanding of brain function changes in BPPV patients, future studies should integrate a broader range of neuroimaging techniques and conduct multi-center cohort studies to validate the results.

## Conclusion

In elderly BPPV patients with anxiety and depression, we identified aberrant hippocampal activity and disrupted hippocampal-cerebellar functional connectivity, suggesting dysfunction within a vestibuli-limbic-cerebellar network. These findings highlight the need for integrated management of psychological symptoms and provide a foundation for developing personalized interventions.

## Data Availability

The datasets used and/or analyzed during the current study are available from the corresponding author on reasonable request.

## Abbreviations

BPPV: Benign paroxysmal positional vertigo
fMRI: Functional magnetic resonance imaging
rs-fMRI: Resting-state functional MRI
ALFF: Amplitude of low-frequency fluctuations
FC: Functional connectivity
HCs: Healthy controls
ROC: Receiver Operating Characteristic
DR: Residual dizziness
CSD: Chronic subjective dizziness
PPPD: Persistent positional perceptual dizziness
BOLD: Blood oxygen level-dependent
OP2: Operculum 2
HADS: Hospital Anxiety and Depression Scale
DHI: Dizziness Handicap Inventory
VSI: Vestibular Symptom Index
EPI: Echo plane imaging
DPABI: Data Processing and Analysis for Brain Imaging
SPM12: Statistical Parametric Mapping 12
FFT: Fast Fourier Transform
